# Association of the vitamin D metabolism gene GC and CYP27B1 polymorphisms with cancer susceptibility: a meta-analysis and trial sequential analysis

**DOI:** 10.1042/BSR20190368

**Published:** 2019-09-13

**Authors:** Man Zhu, Zheqiong Tan, Zhenzhao Luo, Hui Hu, Tangwei Wu, Shiqiang Fang, Hui Wang, Zhongxin Lu

**Affiliations:** Department of Medical Laboratory, The Central Hospital of Wuhan, Tongji Medical College, Huazhong University of Science and Technology, Wuhan, Hubei 430014, P.R. China

**Keywords:** cancer susceptibility, CYP27B1, group-specific component, meta-analysis, polymorphism, vitamin D-binding proteins

## Abstract

Nowadays, vitamin D is known to have functions beyond bone formation, including inhibiting angiogenesis and promoting tumor apoptosis. CYP27B1 and group-specific component (GC), the main enzyme responsible for the degradation and transport of active vitamin D, play important role in many cancer-related cellular processes. Relationships between CYP27B1 and GC polymorphisms and cancer susceptibility have been widely investigated, whereas the results are inconsistent. We strictly searched EMBASE, PubMed, Web of Science, WanFang and CNKI electronic databases for relevant studies exploring the associations of GC (rs4588 and rs7041) and CYP27B1 (rs4646537, rs3782130) polymorphisms with cancer risks according to search strategy. Thirty-two studies published in 13 articles involving 15713 cases and 17304 controls were included. Our analyses suggested that rs4588 and rs7041 polymorphisms were significantly associated with overall cancer risk. Stratification analyses of ethnicity indicated that rs4588 polymorphism significantly increased cancer risk in Caucasians and Asians, while rs7041 polymorphism significantly increased cancer risk in Asians. When studies were stratified by cancer type, our results indicated that rs4588 significantly increased the risk of breast cancer and digestive system tumor, but not in prostate cancer and non-small cell lung cancer, while rs7041 significantly increased the risk of non-small cell lung cancer. Above associations were noteworthy findings as evaluated by false-positive report probabilities (FPRPs). There were no associations of rs4646537 and rs3782130 with overall cancer risks. Associations between CYP27B1 and GC polymorphisms and cancer risks were examined, and additional large samples are necessary to validate our results.

## Introduction

Cancer remains a major global burden of public health. According to the GLOBOCAN 2018, there will be an estimated 18.1 million new cancer cases and 9.6 million deaths in 2018 worldwide [1]. Various causes involving a variety of environmental and genetic factors lead to the development of cancer, although the exact mechanism of carcinogenesis has not been fully understood.

Vitamin D is a fat-soluble vitamin that is closely related to health [2]. They have the following three characteristics: (1) they are found in some natural foods; (2) humans store 7-dehydrocholesterol from cholesterol, which can be converted into vitamin D_3_ after exposure to ultraviolet light; (3) proper sunbathing is enough to satisfy the body’s vitamin D need [2]. Vitamin D deficiency is a ubiquitous phenomenon. Nowadays, vitamin D is known to have functions beyond bone formation, including enhancing immune defense [3], inhibiting cell proliferation [4], inhibiting angiogenesis [5], inhibiting cell metastasis [6], and promoting tumor apoptosis [4]. In addition, vitamin D can reduce mortality in several malignancies [7]. Numerous studies have shown that vitamin D deficiency may be the reason why thousands of patients die prematurely from colon, breast, ovarian and other cancers each year [8–10].

Vitamin D is synthesized by a series of reactions catalyzed by many enzymes. CYP2R1 and CYP27A1 are 25-hydroxylase enzymes that first convert pro-vitamin D absorbed from the diet or produced in the skin after exposure to sunlight [11]. Next, CYP27B1, 1a-hydroxylase converts 25(OH)D into 1,25-dihydroxyvitamin D [1,25(OH)_2_D_3_] in the kidney [11]. Both vitamin D metabolites bind to vitamin D-binding proteins, also known as group-specific component (GC), which aid in the transport of vitamin D [11]. Genetic polymorphisms involving the vitamin D pathway may affect its activity, so if vitamin D does play a role in carcinogenesis, it may be associated with cancer.

Recently, genome-wide association studies (GWASs) have identified CYP27B1 and GC polymorphisms significantly associated with 25(OH)D concentrations [12]. The worldwide variation of CYP27B1 gene (Chromosome 12: 58,156,117-58,162,769 reverse strand) and of its polymorphism SNP rs4646537 (Chromosome 12:58157281 forward strand) and SNP rs3782130 (Chromosome 12:58161898 forward strand), and GC gene (Chromosome 4: 72,607,410-72,669,758 reverse strand) and of its polymorphism SNP rs4588 (Chromosome 4:72618323 forward strand) and SNP rs7041 (Chromosome 4:72618334 forward strand) were analyzed with data obtained from the public database 1000 Genomes Phase 3 Browser. According to the 1000 Genomes Project Phase 3 allele frequencies, the minor allele frequency (MAF) for rs4646537 is 4% in the combined population, the MAF for rs3782130 is 35% in the combined population, the MAF for rs4588 is 21% in the combined population, and the MAF for rs7041 is 38% in the combined population. Up to now, two common CYP27B1 polymorphisms (rs4646537, rs3782130) and two common GC polymorphisms (rs4588 and rs7041) were found to be associated with cancer risks, including breast cancer, non-small cell lung cancer, prostate cancer, hepatocellular carcinoma, esophageal cancer, gastric cancer and colorectal cancer. However, the results are inconsistent, probably because of the limited sample size. To better explore the precise relationship, we performed a meta-analysis and trial sequential analysis (TSA) to characterize the associations of GC (rs4588 and rs7041) and CYP27B1 (rs4646537, rs3782130) polymorphisms with cancer susceptibility.

## Materials and methods

### Literature retrieval

We strictly searched EMBASE, PubMed, Web of Science, Wan Fang and CNKI electronic databases (up to 1 December 2018) for relevant studies exploring the associations of GC (rs4588 and rs7041) and CYP27B1 (rs4646537, rs3782130) polymorphisms with cancer risks according to the search strategy (Supplementary Table S1). Four authors (Man Zhu, Zhenzhao Luo, Zheqiong Tan and Hui Wang) independently searched and screened the search.

### Inclusion and exclusion criteria

Enrolled studies should meet the following inclusion criteria: (A) Human-based research; (B) Case–control/cohort studies; (C) Effective data were available to compute odds ratio (OR), 95% confidence interval (CI) and *P*-value; (D) Involved in the associations of GC (rs4588 and rs7041) or CYP27B1 (rs4646537, rs3782130) polymorphisms (at least one polymorphism involved) with cancer risk; (E) The control group met Hardy–Weinberg equilibrium (HWE). When *P*>0.05, the genetic balance of the population genes is indicated, indicating that the data are from the same Mendelian population [13]. In addition, the enrolled studies also need to meet the following exclusion criteria: (A) Case only or non-cancer subject only studies; (B) Duplicate publications; (C) Conference abstracts.

### Data extraction

Two researchers (Tangwei Wu and Hui Hu) independently screened the detailed data from all enrolled studies. The following data were collected: first author name, issuing time, country, ethnicity, type of cancer, control source, genotyping method, numbers of cases and controls.

### Quality assessment

Two researchers (Tangwei Wu and Hui Hu) assessed the quality of each investigation using the quality assessment criteria (Supplementary Table S2), which was derived from previously published meta-analysis of molecular association studies [14]. The quality assessment criteria cover the methodology for the ascertainment of cancer case (0–2 points), case representation (0–2 points), control representation (0–3 points), control selection (0–2 points), genotyping examination (0–2 points), conformity to HWE (0–1 point) and total sample size (0–3 points). Total scores ranged from 0 to 15, and studies with scores >9 points were classified as high quality.

### Statistical analysis

Stata software (Stata, College Station, TX, U.S.A.), version 12.0, was used for statistical analysis. Associations of GC (rs4588 and rs7041) and CYP27B1 (rs4646537, rs3782130) polymorphisms with cancer risks were estimated by OR and 95% CI. Five different genetic models (dominant, recessive, homozygote, heterozygote and allele model) were used in current study. Statistical heterogeneity was counted by Cochrane Q-test and *P*-values, and random-effect model was used if *P*≤0.10 or *I^2^* ≥ 50%, otherwise, fixed-effect model was used. Stratification analysis was performed based on ethnicity, cancer type and the detection method of genotype. Publication bias (Begg’s test and Egger’s test) analyses and sensitivity analyses were used to evaluate the reliability of current study. *P*<0.05 was considered statistically significant. For each significant finding, false-positive report probability (FPRP) analysis was performed using the method reported by Wacholder et al. [15]. We calculated FPRP assuming a prior probability of 0.1 as previously proposed [16]. We set 0.2 as an FPRP threshold and only result with FPRP-value <0.2 was referred as noteworthy [16].

### TSA

The poor effect of systematic or random errors may increase due to sparse data, which may eventually mislead results in meta-analyses [17]. In order to get more comprehensive results, TSA (Copenhagen Trial Unit, Denmark, 2011) was utilized. In our current study, an overall type-I error of 5%, a statistical test power of 80% and a 20% relative risk reduction was set up.

## Results

### Screening process and characteristics of enrolled studies

**Figure 1:**
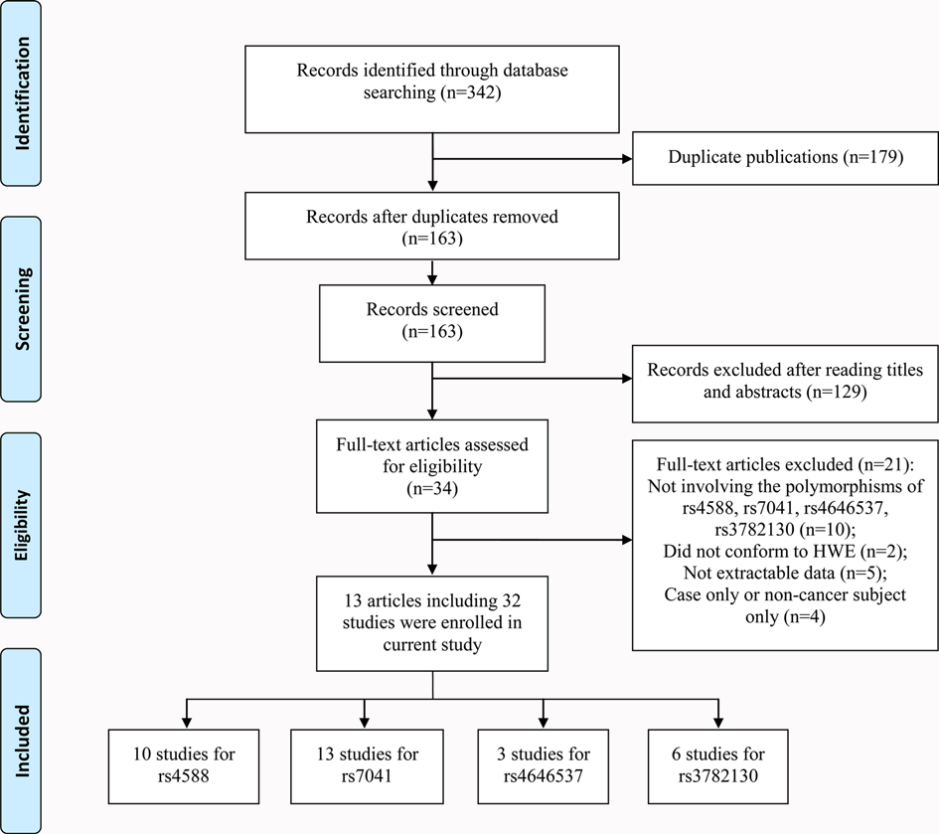
Flow chart of the process for study identification and selection

A total of 342 articles were obtained based on our search strategy. After reading titles and abstracts, 34 articles conformed to our inclusion criteria. After reading full-text, 21 articles were excluded, including 10 that did not describe GC (rs4588 and rs7041) or CYP27B1 (rs4646537, rs3782130) polymorphisms and cancer susceptibility, 2 that did not meet HWE, 4 case only or non-cancer subject only articles, and 5 that not provide detailed genotyping data. Finally, 13 eligible articles including 32 studies (15713 cases and 17304 controls) were enrolled in our current meta-analysis [18–30]. Figure 1 describes the screening process.

In general, sixteen studies included Caucasian populations, fourteen studies included Asian populations and two studies included African populations. TaqMan method was used in nine studies, PCR-RFLP method was used in eighteen studies, Illumina method was used in three studies and two studies used the SNPlex assay method. Ten studies reported the effects of GC polymorphisms in breast cancer, eight reported in digestive system tumor, three in non-small cell lung cancer and two in prostate cancer. Six studies reported the effects of CYP27B1 polymorphisms in prostate cancer, two reported in non-small cell lung cancer and one in digestive system tumor. The characteristics of these studies are listed in Table 1.

**Table 1:** Characteristics of included studies

First author	Year	Country	Ethnicity	Cancer type	Control source	Genotyping method	Cases (AA/AB/BB)	Controls (AA/AB/BB)	HWE	Score
**GC (rs4588)**										
McCullough	2007	U.S.A.	Caucasian	Breast cancer	PB	TaqMan	240/202/48	246/186/44	0.307	12
Anderson	2011	Canada	Caucasian	Breast cancer	PB	PCR-RFLP	792/608/135	846/642/120	0.906	10
Zhou-1	2012	China	Asian	Hepatocellular carcinoma	HB	PCR-RFLP	101/111/25	142/148/25	0.110	7
Zhou-2	2012	China	Asian	Esophageal cancer	HB	PCR-RFLP	148/108/33	159/144/34	0.868	7
Zhou-3	2012	China	Asian	Gastric cancer	HB	PCR-RFLP	74/89/29	88/92/24	0.995	6
Zhou-4	2012	China	Asian	Colorectal cancer	HB	PCR-RFLP	113/100/33	182/134/15	0.117	7
Reimers	2015	U.S.A.	Caucasian	Breast cancer	PB	TaqMan	456/402/82	514/393/84	0.471	10
Deschasaux	2016	France	Caucasian	Breast cancer	PB	TaqMan	101/89/30	227/181/42	0.498	8
Deschasaux	2016	France	Caucasian	Prostate cancer	PB	TaqMan	82/63/20	71/43/10	0.344	7
Wu	2016	China	Asian	Non-small cell lung cancer	PB	PCR-RFLP	235/173/37	230/170/26	0.462	10
**GC (rs7041)**										
McCullough	2007	U.S.A.	Caucasian	Breast cancer	PB	TaqMan	154/237/103	149/235/106	0.460	12
Anderson	2011	Canada	Caucasian	Breast cancer	PB	PCR-RFLP	288/782/558	486/760/309	0.703	10
Zhou-1	2012	China	Asian	Hepatocellular carcinoma	HB	PCR-RFLP	117/98/22	152/139/24	0.311	7
Zhou-2	2012	China	Asian	Esophageal cancer	HB	PCR-RFLP	148/119/22	188/128/21	0.899	7
Zhou-3	2012	China	Asian	Gastric cancer	HB	PCR-RFLP	99/89/16	98/86/10	0.105	6
Zhou-4	2012	China	Asian	Colorectal cancer	HB	PCR-RFLP	123/107/16	171/132/28	0.724	7
Kong	2014	China	Asian	Non-small cell lung cancer	PB	TaqMan	272/339/50	329/240/34	0.254	10
Wang-1	2014	Spain	Caucasian	Breast cancer	PB	Illumina	203/402/221	216/362/201	0.050	13
Wang-2	2014	Non-Spain	Caucasian	Breast cancer	PB	Illumina	42/61/27	73/116/35	0.320	11
Clendenen	2015	Sweden	Caucasian	Breast cancer	PB	Illumina	265/348/121	546/658/229	0.193	9
Reimers	2015	U.S.A.	Caucasian	Breast cancer	PB	TaqMan	239/470/186	311/474/193	0.609	10
Deschasaux	2016	France	Caucasian	Prostate cancer	PB	TaqMan	19/63/45	39/76/50	0.337	7
Wu	2016	China	Asian	Non-small cell lung cancer	PB	PCR-RFLP	173/225/47	175/230/61	0.281	10
**CYP27B1 (rs4646537)**										
Holick	2007	U.S.A.	Caucasian	Prostate cancer	PB	SNPlex assay	546/38/0	497/43/2	0.310	14
Holt-1	2009	U.S.A.	Caucasian	Prostate cancer	PB	PCR-RFLP	319/324/61	314/325/77	0.601	10
Holt-2	2009	U.S.A.	African	Prostate cancer	PB	PCR-RFLP	85/28/2	50/16/1	0.826	7
**CYP27B1 (rs3782130)**										
Holick	2007	U.S.A.	Caucasian	Prostate cancer	PB	SNPlex assay	260/251/75	260/229/61	0.327	14
Holt-1	2009	U.S.A.	Caucasian	Prostate cancer	PB	PCR-RFLP	637/50/2	636/52/0	0.303	10
Holt-2	2009	U.S.A.	African	Prostate cancer	PB	PCR-RFLP	97/15/2	54/8/1	0.298	7
Kong	2014	China	Asian	Non-small cell lung cancer	PB	TaqMan	229/297/77	230/371/120	0.150	10
Mahmoudi	2014	Iran	Asian	Colorectal cancer	HB	PCR-RFLP	144/125/34	180/138/36	0.216	6
Wu	2016	China	Asian	Non-small cell lung cancer	PB	PCR-RFLP	194/149/83	187/163/45	0.300	10

Abbreviations: A, wild type; B, mutated type; HB, hospital-based control; PB, publication-based control.

### Meta-analysis and TSA of rs7041

**Figure 2:**
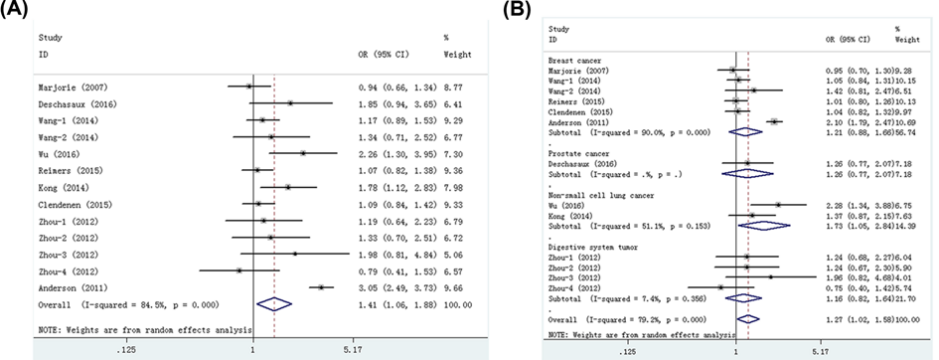
Meta-analysis for the association between rs7041 polymorphism and cancer risk (**A**) Overall comparison (homozygote model); (**B**) stratification analysis by cancer type (recessive model).

Nine publications including thirteen studies with 6916 cases and 7870 controls examined rrs7041 polymorphism. As shown in Table 2, we found that rs7041 polymorphism significantly increased cancer risk in four models: dominant (OR = 1.22, 95% CI = 1.03–1.44, *P*=0.019), recessive (OR = 1.27, 95% CI = 1.02–1.58, *P*=0.030), homozygote (OR = 1.41, 95% CI = 1.06–1.88, *P*=0.017, Figure 2A), and allele (OR = 1.17, 95% CI = 1.02–1.33, *P*=0.022) models. When studies were stratified by ethnicity, significant associations were found in Asians (recessive, OR = 1.40, 95% CI = 1.11–1.77, *P*=0.005; homozygote, OR = 1.52, 95% CI = 1.19–1.93, *P*=0.001; heterozygote, OR = 1.28, 95% CI = 1.00–1.63, *P*=0.047; Allele, OR = 1.20, 95% CI = 1.09–1.32, *P*=0.000). Stratification analyses of cancer type indicated that rs7041 polymorphism increased the risk of non-small cell lung cancer (recessive, OR = 1.73, 95% CI = 1.05–2.84, *P*=0.031, Figure 2B; homozygote, OR = 1.97, 95% CI = 1.38–2.81, *P*=0.000; allele, OR = 1.32, 95% CI = 1.09–1.60, *P*=0.004). Moreover, our data indicated that rs7041 polymorphism was also significantly associated with an increased risk of cancer in the studies with publication-based controls. The FPRP values for significant findings at different prior probability levels are shown in Supplementary Table S3. With the assumption of prior probability of 0.1, these statistically significant associations were noteworthy (FPRP-value <0.2) for overall cancer risk (dominant and allele models), Asians (recessive, homozygote and allele models), non-small cell lung cancer (homozygote and allele models) and PCR-RFLP (heterozygote model) subgroups.

**Table 2:** Meta-analysis of associations between the rs7041 polymorphism and cancer risk

Model	Overall and Stratification analyses	Number of studies	Number of cases/controls	OR (95% CI)	*P*-value	Random/Fixed effect model	*P* for heterogeneity	*I^2^* (%)
Dominant	Overall	13	6916/7870	**1.22 (1.03, 1.44)**	**0.019**	Random	0.000	80.0
	Caucasian	7	4834/5624	1.25 (0.96, 1.63)	0.092	Random	0.000	87.3
	Asian	6	2082/2246	1.19 (0.98, 1.45)	0.077	Random	0.030	59.7
	Breast cancer	6	4707/5459	1.21 (0.92, 1.60)	0.179	Random	0.000	89.2
	Digestive system tumor	4	976/1177	1.08 (0.91, 1.28)	0.364	Fixed	0.811	0
	Non-small cell lung cancer	2	1106/1069	1.38 (0.89, 2.14)	0.150	Random	0.012	84.1
	Prostate cancer	1	127/165	1.76 (0.96, 3.22)	0.067	Fixed	-	-
	PB	9	5940/6693	**1.28 (1.04, 1.59)**	**0.023**	Random	0.000	85.2
	HB	4	976/1177	1.08 (0.91, 1.28)	0.364	Fixed	0.811	0
	PCR-RFLP	6	3049/3198	1.23 (0.90, 1.68)	0.198	Random	0.000	86.2
	TaqMan	4	2177/2236	1.29 (0.95, 1.75)	0.102	Random	0.002	79.3
	Illumina	3	1690/2436	1.11 (0.97, 1.28)	0.119	Fixed	0.796	0
	High quality (>9)	7	5079/5095	1.28 (0.99, 1.64)	0.057	Random	0.000	87.5
	Low quality (≤9)	6	1837/2775	1.11 (0.98, 1.25)	0.104	Fixed	0.653	0
Recessive	Overall	13	6916/7870	**1.27 (1.02, 1.58)**	**0.030**	Random	0.000	79.2
	Caucasian	7	4834/5624	1.21 (0.91, 1.62)	0.192	Random	0.000	88.0
	Asian	6	2082/2246	**1.40 (1.11, 1.77)**	**0.005**	Fixed	0.179	35.2
	Breast cancer	6	4707/5459	1.21 (0.88, 1.66)	0.248	Random	0.000	90.0
	Digestive system tumor	4	976/1177	1.16 (0.84, 1.61)	0.377	Fixed	0.356	7.4
	Non-small cell lung cancer	2	1106/1069	**1.73 (1.05, 2.84)**	**0.031**	Random	0.153	51.1
	Prostate cancer	1	127/165	1.26 (0.77, 2.07)	0.354	Fixed	-	-
	PB	9	5940/6693	**1.30 (1.01, 1.68)**	**0.045**	Random	0.000	85.1
	HB	4	976/1177	1.16 (0.84, 1.61)	0.377	Fixed	0.356	7.4
	PCR-RFLP	6	3049/3198	**1.55 (1.10, 2.19)**	**0.013**	Random	0.017	63.8
	TaqMan	4	2177/2236	1.06 (0.90, 1.24)	0.497	Fixed	0.498	0
	Illumina	3	1690/2436	1.07 (0.91, 1.25)	0.400	Fixed	0.589	0
	High quality (>9)	7	5079/5095	1.35 (0.99, 1.85)	0.055	Random	0.000	87.6
	Low quality (≤9)	6	1837/2775	1.10 (0.92, 1.32)	0.298	Fixed	0.571	0
Homozygote	Overall	13	6916/7870	**1.41 (1.06, 1.88)**	**0.017**	Random	0.000	84.5
	Caucasian	7	4834/5624	1.38 (0.92, 2.07)	0.124	Random	0.000	91.5
	Asian	6	2082/2246	**1.52 (1.19, 1.93)**	**0.001**	Fixed	0.203	31.1
	Breast cancer	6	4707/5459	1.33 (0.85, 2.06)	0.213	Random	0.000	92.9
	Digestive system tumor	4	976/1177	1.19 (0.85, 1.67)	0.315	Fixed	0.420	0
	Non-small cell lung cancer	2	1106/1069	**1.97 (1.38, 2.81)**	**0.000**	Fixed	0.514	0
	Prostate cancer	1	127/165	1.85 (0.94, 3.65)	0.077	Fixed	-	-
	PB	9	5940/6693	**1.49 (1.05, 2.09)**	**0.024**	Random	0.000	89.0
	HB	4	976/1177	1.19 (0.85, 1.67)	0.315	Fixed	0.420	51.1
	PCR-RFLP	6	3049/3198	**1.66 (1.03, 2.69)**	**0.039**	Random	0.000	79.7
	TaqMan	4	2177/2236	1.25 (0.92, 1.69)	0.157	Random	0.078	56
	Illumina	3	1690/2436	1.14 (0.96, 1.37)	0.145	Fixed	0.816	0
	High quality (>9)	7	5079/5095	1.52 (0.99, 2.30)	0.052	Random	0.000	90.7
	Low quality (≤9)	6	1837/2775	1.17 (0.96, 1.43)	0.116	Fixed	0.435	0
Heterozygote	Overall	13	6916/7870	1.18 (0.98, 1.43)	0.081	Random	0.000	68.4
	Caucasian	7	4834/5624	1.14 (0.90, 1.45)	0.279	Random	0.000	79.1
	Asian	6	2082/2246	**1.28 (1.00, 1.63)**	**0.047**	Fixed	0.103	45.3
	Breast cancer	6	4707/5459	1.15 (0.88, 1.49)	0.303	Random	0.000	82.5
	Digestive system tumor	4	976/1177	1.12 (0.80, 1.58)	0.508	Fixed	0.322	14.0
	Non-small cell lung cancer	2	1106/1069	1.52 (0.70, 3.29)	0.285	Random	0.032	78.4
	Prostate cancer	1	127/165	1.09 (0.64, 1.83)	0.758	Fixed	-	-
	PB	9	5940/6693	1.20 (0.96, 1.49)	0.110	Random	0.000	76.6
	HB	4	976/1177	1.12 (0.80, 1.58)	0.508	Fixed	0.322	14.0
	PCR-RFLP	6	3049/3198	**1.48 (1.09, 2.01)**	**0.013**	Random	0.071	50.7
	TaqMan	4	2177/2236	0.99 (0.84, 1.17)	0.904	Fixed	0.975	0
	Illumina	3	1690/2436	1.03 (0.87, 1.21)	0.769	Fixed	0.463	0
	High quality (>9)	7	5079/5095	1.25 (0.96, 1.63)	0.103	Random	0.000	80.6
	Low quality (≤9)	6	1837/2775	1.05 (0.86, 1.27)	0.639	Fixed	0.580	0
Allele	Overall	13	6916/7870	**1.17 (1.02, 1.33)**	**0.022**	Random	0.000	85.2
	Caucasian	7	4834/5624	1.17 (0.95, 1.44)	0.133	Random	0.000	91.7
	Asian	6	2082/2246	**1.20 (1.09, 1.32)**	**0.000**	Fixed	0.137	40.2
	Breast cancer	6	4707/5459	1.15 (0.92, 1.44)	0.217	Random	0.000	93.1
	Digestive system tumor	4	976/1177	1.08 (0.94, 1.23)	0.283	Fixed	0.750	0
	Non-small cell lung cancer	2	1106/1069	**1.32 (1.09, 1.60)**	**0.004**	Random	0.144	53.1
	Prostate cancer	1	127/165	1.33 (0.95, 1.85)	0.096	Fixed	-	-
	PB	9	5940/6693	**1.20 (1.02, 1.42)**	**0.029**	Random	0.000	89.4
	HB	4	976/1177	1.08 (0.94, 1.23)	0.283	Fixed	0.750	0
	PCR-RFLP	6	3049/3198	1.21 (0.95, 1.53)	0.121	Random	0.000	87.0
	TaqMan	4	2177/2236	1.16 (0.96, 1.41)	0.130	Random	0.004	77.6
	Illumina	3	1690/2436	1.07 (0.98, 1.17)	0.128	Fixed	0.919	0
	High quality (>9)	7	5079/5095	1.21 (0.99, 1.48)	0.057	Random	0.000	91.1
	Low quality (≤9)	6	1837/2775	1.08 (0.99, 1.18)	0.086	Fixed	0.728	0

Abbreviations: HB, hospital-based control; PB, publication-based control. Bold values are statistically significant (*P*<0.05).

**Figure 3:**
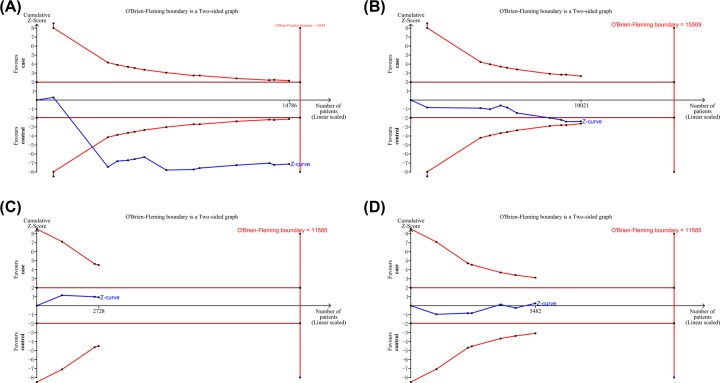
TSAs of the association between rs4588, rs7041, rs3782130 and rs4646537 polymorphisms (dominant model) and cancer risk (**A**) rs7041; (**B**) rs4588; (**C**) rs3782130; (**D**) rs4646537.

As shown in Figure 3A, although the total number of cases did not exceed the O’Brien–Fleming boundary, the cumulative Z-curve exceeded the test sequence monitoring boundary, which verified that rs7041 was significantly associated with cancer susceptibility.

### Meta-analysis and TSA of rs4588

**Figure 4:**
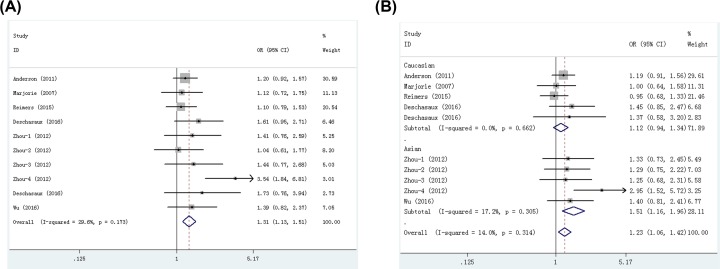
Meta-analysis for the association between rs4588 polymorphism and cancer risk (**A**) Overall comparison (homozygote model); (**B**) stratification analysis by ethnicity (heterozygote model).

Seven publications including ten studies with 4759 cases and 5262 controls examined rs4588 polymorphism. As shown in Table 3, we found that rs4588 polymorphism significantly increased cancer risk in all five models: dominant (OR = 1.10, 95% CI = 1.02–1.19, *P*=0.016), recessive (OR = 1.27, 95% CI = 1.11–1.46, *P*=0.001), homozygote (OR = 1.31, 95% CI = 1.13–1.51, *P*=0.000, Figure 4A), heterozygote (OR = 1.23, 95% CI = 1.06–1.42, *P*=0.005), and allele (OR = 1.11, 95% CI = 1.05–1.18, *P*=0.001) models. Stratification analyses indicated that rs4588 polymorphism significantly increased cancer risk in Caucasians (dominant, OR = 1.10, 95% CI = 1.01–1.21, *P*=0.040; recessive, OR = 1.17, 95% CI = 1.00–1.39, *P*=0.049; homozygote, OR = 1.22, 95% CI = 1.02–1.45, *P*=0.026; allele, OR = 1.10, 95% CI = 1.02–1.18, *P*=0.015) and Asians (recessive, OR = 1.51, 95% CI = 1.18–1.94, *P*=0.001; homozygote, OR = 1.56, 95% CI = 1.06–2.29, *P*=0.024; heterozygote, OR = 1.51, 95% CI = 1.16–1.96, *P*=0.002, Figure 4B). When studies were stratified by cancer type, significant associations were found in breast cancer (dominant, OR = 1.10, 95% CI = 1.00–1.21, *P*=0.046; homozygote, OR = 1.20, 95% CI = 1.00–1.43, *P*=0.047; allele, OR = 1.09, 95% CI = 1.01–1.17, *P*=0.030) and digestive system tumor (recessive, OR = 1.58, 95% CI = 1.02–2.46, *P*=0.042; heterozygote, OR = 1.54, 95% CI = 1.15–2.08, *P*=0.004), but not in prostate cancer and non-small cell lung cancer. Moreover, when studies were stratified by quality score, an increased cancer risk was observed in high quality subgroup in all five genetic models. When studies were stratified by control source and genotyping method, significant associations were found in publication-based controls, hospital-based controls and PCR-RFLP method, but not in TaqMan method. The FPRP values for significant findings at different prior probability levels are shown in Supplementary Table S4. With the assumption of prior probability of 0.1, these statistically significant associations were noteworthy for overall cancer risk (in all five models), Caucasians (homozygote and allele models), Asians (recessive and heterozygote models), digestive system tumor (heterozygote model), breast cancer (allele model) publication-based controls (homozygote and allele models), PCR-RFLP (recessive, homozygote, heterozygote and allele models) and high quality (in all five models) subgroups.

**Table 3:** Meta-analysis of associations between the rs4588 polymorphism and cancer risk

Model	Overall and Stratification analyses	Number of studies	Number of cases/controls	OR (95% CI)	*P*-value	Random/Fixed effect model	*P* for heterogeneity	*I^2^* (%)
Dominant	Overall	10	4759/5262	**1.10 (1.02, 1.19)**	**0.016**	Fixed	0.614	0
	Caucasian	5	3350/3649	**1.10 (1.01, 1.21)**	**0.040**	Fixed	0.770	0
	Asian	5	1409/1613	1.10 (0.95, 1.27)	0.214	Fixed	0.248	26.0
	Breast cancer	4	3185/3525	**1.10 (1.00, 1.21)**	**0.046**	Fixed	0.791	0
	Digestive system tumor	4	964/1187	1.12 (0.94, 1.32)	0.210	Fixed	0.154	42.9
	Prostate cancer	1	165/124	1.36 (0.85, 2.17)	0.203	Fixed	-	-
	Non-small cell lung cancer	1	445/426	1.05 (0.80, 1.37)	0.727	Fixed	-	-
	PB	6	3795/4075	**1.10 (1.00, 1.20)**	**0.040**	Fixed	0.857	0
	HB	4	964/1187	1.12 (0.94, 1.32)	0.210	Fixed	0.154	42.9
	PCR-RFLP	6	2944/3221	1.07 (0.97, 1.18)	0.202	Fixed	0.342	11.4
	TaqMan	4	1815/2041	1.16 (0.98, 1.32)	0.083	Fixed	0.899	0
	High quality (>9)	4	3410/3501	**1.16 (1.02, 1.32)**	**0.047**	Fixed	0.314	15.5
	Low quality (≤9)	6	1349/1761	1.08 (0.98, 1.19)	0.109	Fixed	0.859	0
Recessive	Overall	10	4759/5262	**1.27 (1.11, 1.46)**	**0.001**	Fixed	0.204	26.1
	Caucasian	5	3350/3649	**1.17 (1.00, 1.39)**	**0.049**	Fixed	0.652	0
	Asian	5	1409/1613	**1.51 (1.18, 1.94)**	**0.001**	Fixed	0.128	44.1
	Breast cancer	4	3185/3525	1.16 (0.98, 1.37)	0.092	Fixed	0.588	0
	Digestive system tumor	4	964/1187	**1.58 (1.02, 2.46)**	**0.042**	Random	0.070	57.5
	Prostate cancer	1	165/124	1.57 (0.71, 3.49)	0.266	Fixed	-	-
	Non-small cell lung cancer	1	445/426	1.40 (0.83, 2.35)	0.210	Fixed	-	-
	PB	6	3795/4075	**1.19 (1.02, 1.40)**	**0.029**	Fixed	0.724	0
	HB	4	964/1187	**1.58 (1.02, 2.46)**	**0.042**	Random	0.070	57.5
	PCR-RFLP	6	2944/3221	**1.35 (1.13, 1.61)**	**0.001**	Fixed	0.121	42.6
	TaqMan	4	1815/2041	1.16 (0.93, 1.44)	0.189	Fixed	0.488	0
	High quality (>9)	4	3410/3501	**1.55 (1.23, 1.96)**	**0.000**	Fixed	0.216	29.2
	Low quality (≤9)	6	1349/1761	1.14 (0.96, 1.36)	0.121	Fixed	0.758	0
Homozygote	Overall	10	4759/5262	**1.31 (1.13, 1.51)**	**0.000**	Fixed	0.173	29.6
	Caucasian	5	3350/3649	**1.22 (1.02, 1.45)**	**0.026**	Fixed	0.683	0
	Asian	5	1409/1613	**1.56 (1.06, 2.29)**	**0.024**	Random	0.072	53.4
	Breast cancer	4	3185/3525	**1.20 (1.00, 1.43)**	**0.047**	Fixed	0.671	0
	Digestive system tumor	4	964/1187	1.62 (0.98, 2.68)	0.061	Random	0.037	64.5
	Prostate cancer	1	165/124	1.73 (0.76, 3.94)	0.191	Fixed	-	-
	Non-small cell lung cancer	1	445/426	1.39 (0.82, 2.38)	0.224	Fixed	-	-
	PB	6	3795/4075	**1.23 (1.05, 1.45)**	**0.013**	Fixed	0.775	0
	HB	4	964/1187	1.62 (0.98, 2.68)	0.061	Random	0.037	64.5
	PCR-RFLP	6	2944/3221	**1.45 (1.08, 1.94)**	**0.014**	Random	0.072	50.5
	TaqMan	4	1815/2041	1.23 (0.98, 1.54)	0.077	Fixed	0.518	0
	High quality (>9)	4	3410/3501	**1.59 (1.25, 2.04)**	**0.000**	Fixed	0.130	41.3
	Low quality (≤9)	6	1349/1761	1.18 (0.99, 1.40)	0.069	Fixed	0.892	0
Heterozygote	Overall	10	4759/5262	**1.23 (1.06, 1.42)**	**0.005**	Fixed	0.314	14.0
	Caucasian	5	3350/3649	1.12 (0.94, 1.34)	0.203	Fixed	0.662	0
	Asian	5	1409/1613	**1.51 (1.16, 1.96)**	**0.002**	Fixed	0.305	17.2
	Breast cancer	4	3185/3525	1.11 (0.93, 1.33)	0.251	Fixed	0.534	0
	Digestive system tumor	4	964/1187	**1.54 (1.15, 2.08)**	**0.004**	Fixed	0.191	36.8
	Prostate cancer	1	165/124	1.37 (0.58, 3.20)	0.474	Fixed	-	-
	Non-small cell lung cancer	1	445/426	1.40 (0.81, 2.41)	0.227	Fixed	-	-
	PB	6	3795/4075	1.15 (0.97, 1.35)	0.113	Fixed	0.704	0
	HB	4	964/1187	1.24 (0.99, 1.65)	0.127	Fixed	0.191	36.8
	PCR-RFLP	6	2944/3221	**1.34 (1.12, 1.62)**	**0.002**	Fixed	0.279	20.5
	TaqMan	4	1815/2041	1.07 (0.85, 1.35)	0.546	Fixed	0.552	0
	High quality (>9)	4	3410/3501	**1.51 (1.18, 1.93)**	**0.001**	Fixed	0.438	0
	Low quality (≤9)	6	1349/1761	1.11 (0.92, 1.32)	0.272	Fixed	0.593	0
Allele	Overall	10	4759/5262	**1.11 (1.05, 1.18)**	**0.001**	Fixed	0.284	17.3
	Caucasian	5	3350/3649	**1.10 (1.02, 1.18)**	**0.015**	Fixed	0.685	0
	Asian	5	1409/1613	1.15 (0.99, 1.35)	0.077	Random	0.086	50.9
	Breast cancer	4	3185/3525	**1.09 (1.01, 1.17)**	**0.030**	Fixed	0.764	0
	Digestive system tumor	4	964/1187	1.18 (0.95, 1.45)	0.131	Random	0.049	61.8
	Prostate cancer	1	165/124	1.33 (0.92, 1.93)	0.127	Fixed	-	-
	Non-small cell lung cancer	1	445/426	1.09 (0.88, 1.35)	0.425	Fixed	-	-
	PB	6	3795/4075	**1.10 (1.02, 1.17)**	**0.010**	Fixed	0.809	0
	HB	4	964/1187	1.18 (0.95, 1.45)	0.131	Random	0.049	61.8
	PCR-RFLP	6	2944/3221	**1.10 (1.02, 1.19)**	**0.014**	Fixed	0.104	45.3
	TaqMan	4	1815/2041	1.13 (0.97, 1.25)	0.087	Random	0.085	50.6
	High quality (>9)	4	3410/3501	**1.19 (1.07, 1.33)**	**0.001**	Fixed	0.137	40.0
	Low quality (≤9)	6	1349/1761	1.08 (0.99, 1.16)	0.054	Fixed	0.988	0

Abbreviations: HB, hospital-based control; PB, publication-based control. Bold values are statistically significant (*P*<0.05).

To analyze the reliability of our results, we performed a TSA. As shown in Figure 3B, the cumulative number of cases did not meet the O’Brien–Fleming boundary and test sequence monitoring boundary. Current TSA results suggested that more sample size was still needed for more robust results.

### Meta-analysis and TSA of rs4646537 and rs3782130

Two publications including three studies with 1403 cases and 1325 controls examined rs4646537 polymorphism; five publications including six studies with 2721 cases and 2761 controls examined rs3782130 polymorphism. As shown in Supplementary Table S5, we found these two polymorphisms were not associated with cancer risk.

As for rs4646537 and rs3782130, the cumulative number of cases did not exceed the O’Brien–Fleming boundary and test sequence monitoring boundary (Figure 3C,D). Therefore, more sample sizes were still needed for more robust results.

### Publication bias and sensitivity analysis

As showed in Supplementary Figure S1 and Table 4, Begg’s and Egger’s tests indicated that there was no evidence of significant publication bias in our current meta-analysis. Sensitivity analysis found that none of the single study significantly changed the final conclusion (Supplementary Figure S2).

**Table 4:** Begg’s and Egger’s tests for publication bias

Model	rs4588		rs7041		rs4646537		rs3782130	
	*P*_Begg_	*P*_Egger_	*P*_Begg_	*P*_Egger_	*P*_Begg_	*P*_Egger_	*P*_Begg_	*P*_Egger_
Dominant	0.669	0.573	0.502	0.221	0.602	0.838	0.707	0.727
Recessive	0.132	0.119	0.200	0.498	0.546	0.588	0.310	0.945
Homozygote	0.231	0.124	0.161	0.362	0.573	0.597	0.452	0.833
Heterozygote	0.107	0.132	0.127	0.722	1.000	0.562	0.348	0.736
Allele	0.208	0.130	0.200	0.166	0.609	0.721	0.851	0.947

## Discussion

It has long been clear that genetics has the ability to intervene in the cancer risk in the coming decades. Since polymorphism is the most important cause of human genetic material and information variation, the specific relationship between polymorphisms and cancer susceptibility has attracted widespread attention. With the rapid development of medical science and technology, the field of tumor genetic susceptibility has gradually attracted great interest, and the research on tumor genetic polymorphism is also increasing. Genetic polymorphisms involving the vitamin D pathway has become an important class of genes in the extensive study of polymorphisms in risk factors associated with malignant tumors.

CYP27B1 and GC are two important enzymes involved in vitamin D binding and transport. Nowadays, a growing body of evidence suggests that differential expression of CYP27B1 and GC may play an important role in carcinogenesis development. Reduced CYP27B1 gene expression level has been found in various tumors, including prostate cancer [31–32], non-small cell lung cancer [23]. Whitlatch et al. [32] investigated CYP27B1 expression in normal prostate, prostatic hyperplasia and prostate cancer, and they found that normal prostate exhibited the highest expression of CYP27B1, while its expression was decreased in the following order: prostatic hyperplasia and prostate cancer. These findings suggest that the malignant progression of prostate tissue certainly reduces CYP27B1 expression. Furthermore, Kong et al. [23] found that non-small cell lung cancer patients with high CYP27B1 expression had better overall survival than those with low CYP27B1, which indicated that low CYP27B1 expression was also correlated with a poorer prognosis. In addition, there are two common single nucleotide polymorphisms (rs7041 and rs4588) in GC gene. In the previous reports, genetic variants in the GC gene, including rs7041 and rs4588, have been investigated in breast cancer [18–19,22,25], non-small cell lung cancer [21], prostate cancer [26] and digestive system tumor [27]. However, to date, there is no systematic evaluation on how CYP27B1 and GC polymorphisms are involved in development of cancers.

Our data found that rs4588 was significantly associated with an increased risk of cancer susceptibility, and current result was confirmed by FPRP and TSA analyses. Among these studies, there were four studies on breast cancer, four on digestive system tumor, one on prostate cancer and one on non-small cell lung cancer. Stratified analyses by cancer type revealed a significant association between rs4588 and breast cancer and digestive system tumor, but not in prostate cancer and non-small cell lung cancer. However, our outcomes were different from the results shown by Anderson et al. [18], McCullough et al. [19], Reimers et al. [22], and Deschasaux et al. [25], who demonstrated that rs4588 polymorphism was not associated with breast cancer. This discrepancy may be caused by the limited sample size. Anderson et al. [18] included only 3143 subjects (1535 cases and 1608 controls), McCullough et al. [19] included only 966 subjects (490 cases and 476 controls), Reimers et al. [22] included only 1931 subjects (940 cases and 991 controls), Deschasaux et al. [25] included only 670 subjects (220 cases and 450 controls), which may lack sufficient power to support or deny an association. Previous studies also focused on the relationship between the rs4588 and digestive system tumor. However, our outcomes were different from previous study [27], which indicated that rs4588 polymorphism was not associated with hepatocellular carcinoma, esophageal cancer and gastric cancer. Possible reasons for this difference could be explained as the limited sample size. There was only one study for hepatocellular carcinoma, esophageal cancer and gastric cancer, which was far from enough to obtain trustworthy results. Based on current TSA results, more studies by standardized unbiased methods are required to offer more detailed data.

As for rs7041, we found that this polymorphism significantly increased cancer risk. Stratification analyses of ethnicity suggested rs7041 increased cancer risk in Asians, but not in Caucasians. Possible reasons can be explained as the different genetic backgrounds of cancer across ethnicities. In this meta-analysis, the pooled rs7041 C allele frequency of the controls showed a large difference across ethnicities (Asians: 30.2%; Caucasians: 45.4%), which may possibly affect the relationships between rs7041 polymorphism and cancer risk among different racial subgroups. Moreover, when studies were stratified by cancer type, we also found that rs7041 polymorphism was significantly associated with an increased risk in the non-small cell lung cancer. However, most subgroups had insufficient numbers, which may attenuate the statistical power. Our results were partially consistent with the consequence of the study by Wang et al. [20], which reported that there was no significant association between rs7041 and breast cancer in Asians and Caucasians. However, study by Reimers et al [22]. suggested that rs7041 was associated with an increased risk of breast cancer in Caucasians. It is noteworthy that Yao et al. [33] indicated that increased polymorphism may be related to the higher prevalence of estrogen receptor (ER)-negative but not ER-positive breast cancer. At present, a large number of researches indicated that there were important differences in genetic susceptibility between ER-negative and ER-positive breast cancer [11]. Therefore, it is reasonable to hypothesize that rs7041 polymorphism may have a specific effect on the susceptibility to ER-negative breast cancer. Of note, due to limited data, lack of further evaluation between rs7041 and ER-negative and ER-positive breast cancer prevented our comprehensive understanding. Further large-cohort and well-designed studies are necessary to identify the possible association between them. With respect to the remaining two polymorphisms, we failed to find any associations between rs4646537 and rs3782130 and cancer risk. Given the limited sample size, our results should be interpreted with caution.

In general, current analysis has the following advantages: (1) Our research results were validated based on TSA to ensure the reliability of the results. (2) All included studies were consistent with the HWE balance law, which may improve the reliability of our study. (3): This system evaluation is the first analysis of reviewing the relationships between CYP27B1 (rs4646537, rs3782130) and GC (rs4588 and rs7041) polymorphisms with cancer susceptibility. (4) To avoid false positive findings, FPRP analyses were used for all significant findings observed in our study. However, current study still has the following shortcomings: (1) The subjects we included were limited to Caucasians and Asians, and the results of the present study still lack information from other ethnic groups, which may lead to publication bias. (2) The number of studies on rs4646537, rs3782130, rs4588 and rs7041 was relatively small in some subgroups, which may create significant or insignificant results by chance. (3) In some included studies, detailed information (e.g., radiation exposure, carcinogen, smoking and other risk factors) was not gathered, which further prevented the stratification analyses. Thus, a larger sample size, multi-racial, multi-center standardized research is needed to provide more detailed data in the future.

## Conclusions

In conclusion, this systematical meta-analysis indicated that rs4588 and rs7041 polymorphisms play important roles in cancer pathogenesis, especially in non-small cell lung cancer, breast cancer and digestive system tumor, which were noteworthy findings as evaluated by FPRP. However, the other two polymorphisms (rs4646537 and rs3782130) are not associated with cancer risk. Further well-designed studies are necessary to validate our results.

## Supporting information

**Supplementary Figure S1:** Begg’s test for publication bias (dominant model) **A** rs4588; **B** rs7041; **C** rs3782130; **D** rs4646537

**Supplementary Figure S2:** Sensitivity analyses of the studies (allele model) **A** rs4588; **B** rs7041; **C** rs4646537; **D** rs3782130

**Supplementary Table S1:** The detailed search strategies of the associations between GC (rs4588, rs7041), CYP27B1 (rs4646537, and rs3782130) polymorphisms and cancer risk

**Supplementary Table S2:** Score of quality assessment

**Supplementary Table S3:** False-positive report probability values for associations between the rs7041 polymorphism and cancer risk

**Supplementary Table S4:** False-positive report probability values for associations between the rs4588 polymorphism and cancer risk

**Supplementary Table S5:** Meta-analysis of associations between rs4646537 and rs3782130 polymorphisms and cancer risk

## Abbreviations

CI: confidence interval
CNKI: China national knowledge infrastructure
EMBASE: excerpta medica database
ER: estrogen receptor
FPRP: false-positive report probability
GC: group-specific component
HWE: Hardy–Weinberg equilibrium
MAF: minor allele frequency
OR: odds ratio
PCR-RFLP: polymerase chain reaction-restrictionfragment length polymorphism
SNP: single nucleotide polymorphism
TSA: trial sequential analysis

## References

[1] Bray F., Ferlay J., Soerjomataram I. (2018) Global cancer statistics 2018: GLOBOCAN estimates of incidence and mortality worldwide for 36 cancers in 185 countries. CA Cancer J. Clin. 68, 394–424 10.3322/caac.21492 30207593

[2] Jukic A.M.Z., Hoofnagle A.N. and Lutsey P.L. (2018) Measurement of vitamin D for epidemiologic and clinical research: shining light on a complex decision. Am. J. Epidemiol. 187, 879–890 10.1093/aje/kwx297 29020155 PMC5889008

[3] Abdo J., Rai V. and Agrawal D.K. (2017) Interplay of immunity and vitamin D: interactions and implications with current IBD therapy. Curr. Med. Chem. 24, 852–867 10.2174/0929867323666161026124951 27784213

[4] Gao Y., Um C.Y., Fedirko V. (2018) Effects of supplemental vitamin D and calcium on markers of proliferation, differentiation, and apoptosis in the normal colorectal mucosa of colorectal adenoma patients. PLoS ONE 13, e0208762 10.1371/journal.pone.0208762 30557404 PMC6296527

[5] Berger M.D., Stintzing S., Heinemann V. (2018) A polymorphism within the vitamin D transporter gene predicts outcome in metastatic colorectal cancer patients treated with FOLFIRI/Bevacizumab or FOLFIRI/Cetuximab. Clin. Cancer Res. 24, 784–793 10.1158/1078-0432.CCR-17-1663 29208668 PMC7505162

[6] Chiang K.C., Yeh T.S., Chen S.C. (2016) The vitamin D analog, MART-10, attenuates triple negative breast cancer cells metastatic potential. Int. J. Mol. Sci. 17, E606 10.3390/ijms17040606 27110769 PMC4849057

[7] Brenner H., Jansen L., Saum K.U. (2017) Vitamin D supplementation trials aimed at reducing mortality have much higher power when focusing on people with low serum 25-Hydroxyvitamin D concentrations. J. Nutr. 147, 1325–1333 10.3945/jn.117.250191 28539415

[8] de La Puente-Yagüe M., Cuadrado-Cenzual M.A., Ciudad-Cabañas M.J. (2018) Vitamin D: And its role in breast cancer. Kaohsiung J. Med. Sci. 34, 423–427 10.1016/j.kjms.2018.03.004 30041759 PMC11915700

[9] Cho Y.A., Lee J., Oh J.H. (2018) Vitamin D receptor FokI polymorphism and the risks of colorectal cancer, inflammatory bowel disease, and colorectal adenoma. Sci. Rep. 8, 12899 10.1038/s41598-018-31244-5 30150667 PMC6110797

[10] Ong J.S., Gharahkhani P., An J. (2018) Vitamin D and overall cancer risk and cancer mortality: a Mendelian randomization study. Hum. Mol. Genet. 27, 4315–4322 30508204 10.1093/hmg/ddy307

[11] Zhu M., Qiu S., Zhang X. (2018) The associations between CYP24A1 polymorphisms and cancer susceptibility: a meta-analysis and trial sequential analysis. Pathol. Res. Pract. 214, 53–63 10.1016/j.prp.2017.11.014 29254801

[12] Wang S., Huo D., Kupfer S. (2018) Genetic variation in the vitamin D related pathway and breast cancer risk in women of African ancestry in the root consortium. Int. J. Cancer 142, 36–43 10.1002/ijc.31038 28891071 PMC5755399

[13] Meng J., Wang S., Zhang M. (2018) TP73 G4C14-A4T14 polymorphism and cancer susceptibility: evidence from 36 case-control studies. Biosci. Rep. 38, BSR20181452 10.1042/BSR20181452 30420492 PMC6294616

[14] He J., Liao X.Y., Zhu J.H. (2014) Association of MTHFR C677T and A1298C polymorphisms with non-Hodgkin lymphoma susceptibility: evidence from a meta-analysis. Sci. Rep. 4, 6159 10.1038/srep06159 25146845 PMC5381410

[15] Wacholder S., Chanock S., Garcia-Closas M. (2004) Assessing the probability that a positive report is false: an approach for molecular epidemiology studies. J. Natl. Cancer Inst. 96, 434–442 10.1093/jnci/djh075 15026468 PMC7713993

[16] He J., Wang M.Y., Qiu L.X. (2013) Genetic variations of mTORC1 genes and risk of gastric cancer in an Eastern Chinese population. Mol. Carcinog. 52, E70–E79 10.1002/mc.22013 23423739

[17] Khan S., Dar S.A., Mandal R.K. (2018) Angiotensin-converting enzyme gene I/D polymorphism is associated with systemic lupus erythematosus susceptibility: an updated meta-analysis and trial sequential analysis. Front. Physiol. 9, 1793 10.3389/fphys.2018.01793 30618805 PMC6305102

[18] Anderson L.N., Cotterchio M., Cole D.E. (2011) Vitamin D-related genetic variants, interactions with vitamin D exposure, and breast cancer risk among Caucasian women in Ontario. Cancer Epidemiol. Biomark. Prev. 20, 1708–1717 10.1158/1055-9965.EPI-11-0300 21693626

[19] McCullough M.L., Stevens V.L., Diver W.R. (2007) Vitamin D pathway gene polymorphisms, diet, and risk of postmenopausal breast cancer: a nested case-control study. Breast Cancer Res. 9, 9 10.1186/bcr1642 PMC1851389 17244366

[20] Wang W., Ingles S.A., Torres-Mejía G. (2014) Genetic variants and non-genetic factors predict circulating vitamin D levels in Hispanic and non-Hispanic White women: the Breast Cancer Health Disparities Study. Int. J. Mol. Epidemiol. Genet 5, 31–46 24596595 PMC3939005

[21] Wu X., Cheng J., Yang K. (2016) Vitamin D-related gene polymorphisms, plasma 25-Hydroxy-Vitamin D, Cigarette smoke and non-small cell lung cancer (NSCLC) risk. Int. J. Mol. Sci. 17, E1597 10.3390/ijms17101597 27669215 PMC5085630

[22] Reimers L.L., Crew K.D., Bradshaw P.T. (2015) Vitamin D-related gene polymorphisms, plasma 25-hydroxyvitamin D, and breast cancer risk. Cancer Causes Control 26, 187–203 10.1007/s10552-014-0497-9 25421379 PMC4302042

[23] Kong J., Xu F., Qu J. (2015) Genetic polymorphisms in the vitamin D pathway in relation to lung cancer risk and survival. Oncotarget 6, 2573–2582 10.18632/oncotarget.2951 25544771 PMC4385872

[24] Clendenen T.V., Ge W., Koenig K.L. (2015) Genetic polymorphisms in vitamin D metabolism and signaling genes and risk of breast cancer: a nested case-control study. PLoS ONE 10, e0140478 10.1371/journal.pone.0140478 26488576 PMC4619526

[25] Deschasaux M., Souberbielle J.C., Latino-Martel P. (2016) Weight status and alcohol intake modify the association between vitamin D and breast cancer risk. J. Nutr. 146, 576–585 10.3945/jn.115.221481 26817718

[26] Deschasaux M., Souberbielle J.C., Latino-Martel P. (2016) A prospective study of plasma 25-hydroxyvitamin D concentration and prostate cancer risk. Br. J. Nutr. 115, 305–314 10.1017/S0007114515004353 26568368

[27] Zhou L., Zhang X., Chen X. (2012) GC Glu416Asp and Thr420Lys polymorphisms contribute to gastrointestinal cancer susceptibility in a Chinese population. Int. J. Clin. Exp. Med. 5, 72–79 22328951 PMC3272689

[28] Holick C.N., Stanford J.L., Kwon E.M. (2007) Comprehensive association analysis of the vitamin D pathway genes, VDR, CYP27B1, and CYP24A1, in prostate cancer. Cancer Epidemiol. Biomark. Prev. 16, 1990–1999 10.1158/1055-9965.EPI-07-0487 17932346

[29] Holt S.K., Kwon E.M., Peters U. (2009) Vitamin D pathway gene variants and prostate cancer risk. Cancer Epidemiol. Biomark. Prev. 18, 1929–1933 10.1158/1055-9965.EPI-09-0113 19454612 PMC2743676

[30] Mahmoudi T., Karimi K., Arkani M. (2014) Lack of associations between Vitamin D metabolism-related gene variants and risk of colorectal cancer. Asian Pac. J. Cancer Prev. 15, 957–961 10.7314/APJCP.2014.15.2.957 24568525

[31] Susa T., Iizuka M., Okinaga H. (2018) Without 1α-hydroxylation, the gene expression profile of 25(OH)D3 treatment overlaps deeply with that of 1,25(OH)_2_D_3_ in prostate cancer cells. Sci. Rep. 8, 9024 10.1038/s41598-018-27441-x 29899561 PMC5998076

[32] Whitlatch L.W., Young M.V., Schwartz G.G. (2002) 25-Hydroxyvitamin D-1alpha-hydroxylase activity is diminished in human prostate cancer cells and is enhanced by gene transfer. J. Steroid Biochem. Mol. Biol. 81, 135–140 10.1016/S0960-0760(02)00053-5 12137802

[33] Yao S., Zirpoli G., Bovbjerg D.H. (2012) Variants in the vitamin D pathway, serum levels of vitamin D, and estrogen receptor negative breast cancer among African-American women: a case-control study. Breast Cancer Res. 14, 58 10.1186/bcr3162 PMC3446393 22480149

