# Supplementary material for: Association of the vitamin D metabolism gene GC and CYP27B1 polymorphisms with cancer susceptibility: a meta-analysis and trial sequential analysis
Source: Biosci Rep. 2019 Sep 13;39(9):BSR20190368. doi: 10.1042/BSR20190368 (PMC6744587; doi:10.1042/BSR20190368)
Supplement: Supplementary file 1 [file bsr20190368_Supp1.pdf]

Supplementary Materials

Supplementary Tables

Table S1 The detailed search strategies of the associations between GC (rs4588, rs7041), CYP27B1 (rs4646537, and rs3782130) polymorphisms and cancer risk

Database	Search strategy	
Pubmed	#1: Polymorphism, genetic<br>#2: Polymorphism*<br>#3: SNP<br>#4: Single nucleotide polymorphism<br>#5: Variant<br>#6: Mutation<br>#7: #1 OR #2 OR #3 OR #4 OR #5 OR #6<br>#8: rs4588<br>#9: rs7041<br>#10: rs4646537<br>#11: rs3782130<br>#12: vitamin D-binding protein<br>#13: CYP27B1<br>#14: #8 OR #9 OR #10 OR #11 OR #12 OR #13<br>#15: Neoplasms<br>#16: Cancer<br>#17: Carcino*<br>#18: #15 OR #16 OR #17<br>#19: #7 AND #14 AND #18	((Polymorphism, genetic) OR Polymorphism* OR SNP OR (Single nucleotide polymorphism) OR Variant OR Mutation) AND (rs4588 OR rs7041 OR rs4646537 OR rs3782130 OR CYP24A1 OR vitamin D-binding protein OR CYP27B1) AND (Neoplasms OR Cancer OR Carcino*))
Embase	#1: 'neoplasm'/exp<br>#2: cancer<br>#3: tumor<br>#4: carcinoma<br>#5: carcinogenesis<br>#6: #1 OR #2 OR #3 OR #4 OR #5<br>#7: rs4588<br>#8: rs7041<br>#9: rs4646537<br>#10: rs3782130<br>#11: vitamin D-binding protein<br>#12: CYP27B1<br>#13: #7 OR #8 OR #9 OR #10 OR #11 OR #12<br>#14: 'single nucleotide polymorphism'/exp<br>#15: SNP	(('neoplasm'/exp OR cancer OR tumor OR carcinoma OR carcinogenesis) AND (rs4588 OR rs7041 OR rs4646537 OR rs3782130 OR CYP24A1 OR vitamin D-binding protein OR CYP27B1) AND ('single nucleotide polymorphism'/exp OR SNP OR polymorphism OR variant OR mutation))

	#16: polymorphism
	#17: variant
	#18: mutation
	#19: #14 OR #15 OR #16 OR #17 OR #18
	#20: #6 AND #13 AND #19

Table S2. Score of quality assessment

Criteria	Score
Representativeness of case	
Selected from population cancer registry	2
Selected from hospital	1
No method of selection described	0
Representativeness of control	
Population-based	3
Blood donors	2
Hospital-based	1
Not described	0
Ascertainment of cancer case	
Histopathologic confirmation	2
By patient medical record	1
Not described	0
Control selection	
Controls matched with cases by age and sex	2
Controls matched with cases only by age or by sex	1
Not matched or not descried	0
Genotyping examination	
Genotyping done blindly and quality control	2
Only genotyping done blindly or quality control	1
Unblinded and without quality control	0
HWE	
HWE in the control group	1
HWD in the control group or not mentioned	0
Total sample size	
> 1000	3
501 - 1000	2
201 - 500	1
≤ 200	0

Table S3. False-positive report probability values for associations between the rs7041 polymorphism and cancer risk

Significant association	OR (95%CI)	*P* [a]	Statistical power [b]	Prior probability				
				0.25	0.1	0.01	0.001	0.0001
**Dominant**								
Overall	1.22(1.03, 1.44)	0.019	0.993	**0.054**	**0.145**	0.651	0.950	0.995
PB	1.28(1.04, 1.59)	0.023	0.924	**0.077**	0.200	0.733	0.965	0.996
**Recessive**								
Overall	1.27(1.02, 1.58)	0.030	0.932	**0.093**	0.236	0.772	0.972	0.997
Asian	1.40(1.11, 1.77)	0.005	0.718	**0.020**	**0.058**	**0.404**	**0.873**	**0.986**
Non-small cell lung cancer	1.73(1.05, 2.84)	0.031	0.286	0.240	0.487	0.913	0.991	0.999
PB	1.30(1.01, 1.68)	0.045	0.863	**0.135**	0.319	0.837	0.981	0.998
PCR-RFLP	1.55(1.10, 2.19)	0.013	0.426	**0.084**	0.215	0.750	0.968	0.997
**Homozygote**								
Overall	1.41(1.06, 1.88)	0.017	0.663	**0.080**	0.207	0.742	0.967	0.997
Asian	1.52(1.19, 1.93)	0.001	0.457	**0.004**	**0.011**	**0.113**	0.563	0.928
Non-small cell lung cancer	1.97(1.38, 2.81)	0.000	0.066	**0.008**	**0.024**	0.214	0.734	0.965
PB	1.49(1.05, 2.09)	0.024	0.515	**0.108**	0.267	0.801	0.976	0.998
PCR-RFLP	1.66(1.03, 2.69)	0.039	0.340	0.259	0.512	0.920	0.991	0.999
**Heterozygote**								
Asian	1.28(1.00, 1.63)	0.047	0.901	**0.131**	0.312	0.833	0.980	0.998
PCR-RFLP	1.48(1.09, 2.01)	0.013	0.534	**0.063**	**0.169**	0.691	0.958	0.996
**Allele**								
Overall	1.17(1.02, 1.33)	0.022	1.000	**0.047**	**0.128**	0.618	0.942	0.994
Asian	1.20(1.09, 1.32)	0.000	1.000	**0.001**	**0.002**	**0.017**	**0.150**	0.639
Non-small cell lung cancer	1.32(1.09, 1.60)	0.004	0.904	**0.015**	**0.044**	0.339	0.838	0.981

PB	1.20(1.02, 1.42)	0.029	0.995	**0.092**	0.234	0.771	0.971	0.997

The results in false-positive report probability analysis were in bold, if the prior probability <0.2. OR: odds ratio; CI: confidence interval; [a] *P* value for significant test; [b] Statistical power was calculated using the number of observations in the meta-analysis and the OR and *P* value in this table.

Table S4. False-positive report probability values for associations between the rs4588 polymorphism and cancer risk

Significant association	OR (95%CI)	*P* [a]	Statistical power [b]	Prior probability				
				0.25	0.1	0.01	0.001	0.0001
**Dominant**								
Overall	1.10(1.02, 1.19)	0.016	1.000	**0.050**	**0.136**	0.634	0.946	0.994
Caucasian	1.10(1.01, 1.21)	0.040	1.000	**0.130**	0.310	0.832	0.980	0.998
Breast cancer	1.10(1.00, 1.21)	0.046	1.000	**0.130**	0.310	0.832	0.980	0.998
PB	1.10(1.00, 1.20)	0.040	1.000	**0.087**	0.223	0.759	0.969	0.997
High quality (> 9)	1.16(1.02, 1.32)	0.047	1.000	**0.068**	**0.180**	0.707	0.961	0.996
**Recessive**								
Overall	1.27(1.11, 1.46)	0.001	0.990	**0.002**	**0.007**	**0.072**	0.440	0.887
Caucasian	1.17(1.00, 1.39)	0.049	0.998	**0.182**	0.401	0.880	0.987	0.999
Asian	1.51(1.18, 1.94)	0.001	0.479	**0.008**	**0.023**	0.207	0.725	0.964
Digestive system tumor	1.58(1.02, 2.46)	0.042	0.409	0.239	0.485	0.912	0.991	0.999
PB	1.19(1.02, 1.40)	0.029	0.997	**0.097**	0.245	0.781	0.973	0.997
HB	1.58(1.02, 2.46)	0.042	0.409	0.239	0.485	0.912	0.991	0.999
PCR-RFLP	1.35(1.13, 1.61)	0.001	0.879	**0.003**	**0.009**	**0.086**	0.488	0.905
High quality (> 9)	1.55(1.23, 1.96)	0.000	0.392	**0.002**	**0.006**	**0.060**	0.391	0.865
**Homozygote**								
Overall	1.31(1.13, 1.51)	0.000	0.969	**0.001**	**0.002**	**0.020**	**0.168**	0.668
Caucasian	1.22(1.02, 1.45)	0.026	0.990	**0.068**	**0.179**	0.706	0.960	0.996
Asian	1.56(1.06, 2.29)	0.024	0.421	**0.142**	0.331	0.845	0.982	0.998
Breast cancer	1.20(1.00, 1.43)	0.047	0.994	**0.111**	0.273	0.805	0.977	0.998
PB	1.23(1.05, 1.45)	0.013	0.991	**0.040**	**0.110**	0.577	0.932	0.993
PCR-RFLP	1.45(1.08, 1.94)	0.014	0.590	**0.059**	**0.159**	0.675	0.954	0.995

High quality (> 9)	1.59(1.25, 2.04)	0.000	0.323	**0.002**	**0.007**	**0.075**	0.450	0.891
**Heterozygote**								
Overall	1.23(1.06, 1.42)	0.005	0.997	**0.014**	**0.041**	0.320	0.826	0.979
Asian	1.51(1.16, 1.96)	0.002	0.480	**0.012**	**0.035**	0.287	0.803	0.976
Digestive system tumor	1.54(1.15, 2.08)	0.004	0.432	**0.033**	**0.092**	0.528	0.918	0.991
PCR-RFLP	1.34(1.12, 1.62)	0.002	0.878	**0.008**	**0.025**	0.220	0.740	0.966
High quality (> 9)	1.51(1.18, 1.93)	0.001	0.479	**0.006**	**0.018**	**0.171**	0.675	0.954
**Allele**								
Overall	1.11(1.05, 1.18)	0.001	1.000	**0.002**	**0.007**	**0.075**	0.451	0.892
Caucasian	1.10(1.02, 1.18)	0.015	1.000	**0.023**	**0.066**	0.436	0.886	0.987
Breast cancer	1.09(1.01, 1.17)	0.030	1.000	**0.049**	**0.133**	0.628	0.945	0.994
PB	1.10(1.02, 1.17)	0.010	1.000	**0.007**	**0.022**	**0.196**	0.711	0.961
PCR-RFLP	1.10(1.02, 1.19)	0.014	1.000	**0.050**	**0.136**	0.634	0.946	0.994
High quality (> 9)	1.19(1.07, 1.33)	0.001	1.000	**0.006**	**0.019**	**0.177**	0.685	0.956

The results in false-positive report probability analysis were in bold, if the prior probability <0.2. OR: odds ratio; CI: confidence interval; [a] *P* value for significant test; [b] Statistical power was calculated using the number of observations in the meta-analysis and the OR and *P* value in this table.

Table S5. Meta-analysis of associations between rs4646537 and rs3782130 polymorphisms and cancer risk

Variables	N	Dominant model	Recessive model	Homozygote model	Heterozygote model	Allele model
		OR(95%CI)/I^2 %/P_Q	OR(95%CI)/I^2 %/P_Q	OR(95%CI)/I^2 %/P_Q	OR(95%CI)/I^2 %/P_Q	OR(95%CI)/I^2 %/P_Q
**rs4646537**						
Overall	3	0.92(0.76, 1.10)/0/0.675	0.77(0.55, 1.09)/0/0.615	0.76(0.53, 1.10)/0/0.610	0.78(0.54, 1.12)/0/0.695	0.91(0.79, 1.05)/0/0.591
Caucasian	2	0.91(0.75, 1.10)/0/0.419	0.77(0.54, 1.09)/0/0.353	0.76(0.52, 1.09)/0/0.351	0.78(0.54, 1.12)/0/0.424	0.90(0.78, 1.04)/0/0.357
African	1	1.04(0.52, 2.07)	1.17(0.10, 13.13)	1.18(0.10, 13.31)	1.14(0.10, 13.62)	1.04(0.56, 1.94)
Prostate cancer	3	0.92(0.76, 1.10)/0/0.675	0.77(0.55, 1.09)/0/0.615	0.76(0.53, 1.10)/0/0.610	0.78(0.54, 1.12)/0/0.695	0.91(0.79, 1.05)/0/0.591
PCR-RFLP	2	0.95(0.78, 1.16)/0/0.793	0.79(0.56, 1.13)/0/0.752	0.79(0.55, 1.14)/0/0.743	0.80(0.56, 1.15)/0/0.776	0.93(0.80, 1.08)/0/0.710
SNPlex assay	1	0.77(0.49, 1.20)	0.19(0.01, 3.86)	0.18(0.01, 3.80)	0.23(0.01, 4.85)	0.74(0.48, 1.15)
High quality (> 9)	2	0.91(0.75, 1.10)/0/0.419	0.77(0.54, 1.09)/0/0.353	0.76(0.52, 1.09)/0/0.351	0.78(0.54, 1.12)/0/0.424	0.90(0.78, 1.04)/0/0.357
Low quality (≤ 9)	1	1.04(0.52, 2.07)	1.17(0.10, 13.13)	1.18(0.10, 13.31)	1.14(0.10, 13.62)	1.04(0.56, 1.94)
**rs3782130**						
Overall	6	0.99(0.87, 1.11)/0/0.533	1.14(0.95, 1.38)/0/0.417	1.11(0.91, 1.35)/0/0.526	1.19(0.97, 1.44)/1.4/0.407	1.02(0.94, 1.12)/0/0.665
Caucasian	2	1.09(0.89, 1.33)/0/0.616	1.21(0.85, 1.73)/0/0.353	1.27(0.87, 1.84)/0/0.369	1.16(0.80, 1.69)/0/0.329	1.10(0.94, 1.29)/0/0.760
African	1	1.05(0.44, 2.52)	1.11(0.10, 12.46)	1.11(0.10, 12.56)	1.07(0.08, 13.65)	1.06(0.47, 2.34)
Asian	3	0.93(0.79, 1.10)/13/0.317	1.13(0.82, 1.55)/50/0.134	1.06(0.84, 1.33)/28/0.251	1.18(0.84, 1.67)/51/0.130	0.99(0.89, 1.11)/3/0.357
Prostate cancer	3	1.09(0.89, 1.33)/0/0.879	1.21(0.85, 1.72)/0/0.648	1.26(0.87, 1.83)/0/0.665	1.16(0.80, 1.68)/0/0.620	1.10(0.94, 1.28)/0/0.950
NSCLC	2	0.87(0.73, 1.04)/0/0.976	1.14(0.70, 1.85)/75/0.045	1.04(0.68, 1.57)/61/0.111	1.24(0.75, 2.05)/73/0.053	0.96(0.85, 1.09)/0/0.331
Colorectal cancer	1	1.14(0.84, 1.55)	1.12(0.68, 1.83)	1.18(0.70, 1.98)	1.04(0.62, 1.77)	1.11(0.88, 1.40)
PCR-RFLP	4	0.98(0.83, 1.17)/0/0.616	0.98(0.74, 1.28)/0/0.638	0.96(0.72, 1.29)/0/0.517	1.01(0.75, 1.36)/0/0.753	0.98(0.86, 1.13)/0/0.558
Other methods	2	0.99(0.77, 1.27)/57/0.128	1.32(0.96, 1.69)/0/0.395	1.26(0.96, 1.65)/0/0.864	1.35(0.94, 1.76)/42/0.189	1.06(0.94, 1.19)/0/0.443
High quality (> 9)	4	0.96(0.84, 1.09)/1/0.387	1.15(0.94, 1.40)/40/0.173	1.10(0.89, 1.36)/27/0.251	1.21(0.98, 1.50)/38/0.186	1.01(0.92, 1.11)/0/0.558
Low quality (≤ 9)	2	1.13(0.85, 1.51)/0/0.861	1.12(0.95, 1.38)/0/0.995	1.18(0.71, 1.95)/0/0.963	1.04(0.62, 1.75)/0/0.986	1.10(0.88, 1.38)/0/0.907

Abbreviation: OR: Odds ratio; CI: Confidence interval; P_Q: P value of the Q-test for heterogeneity test.

Supplementary Figures

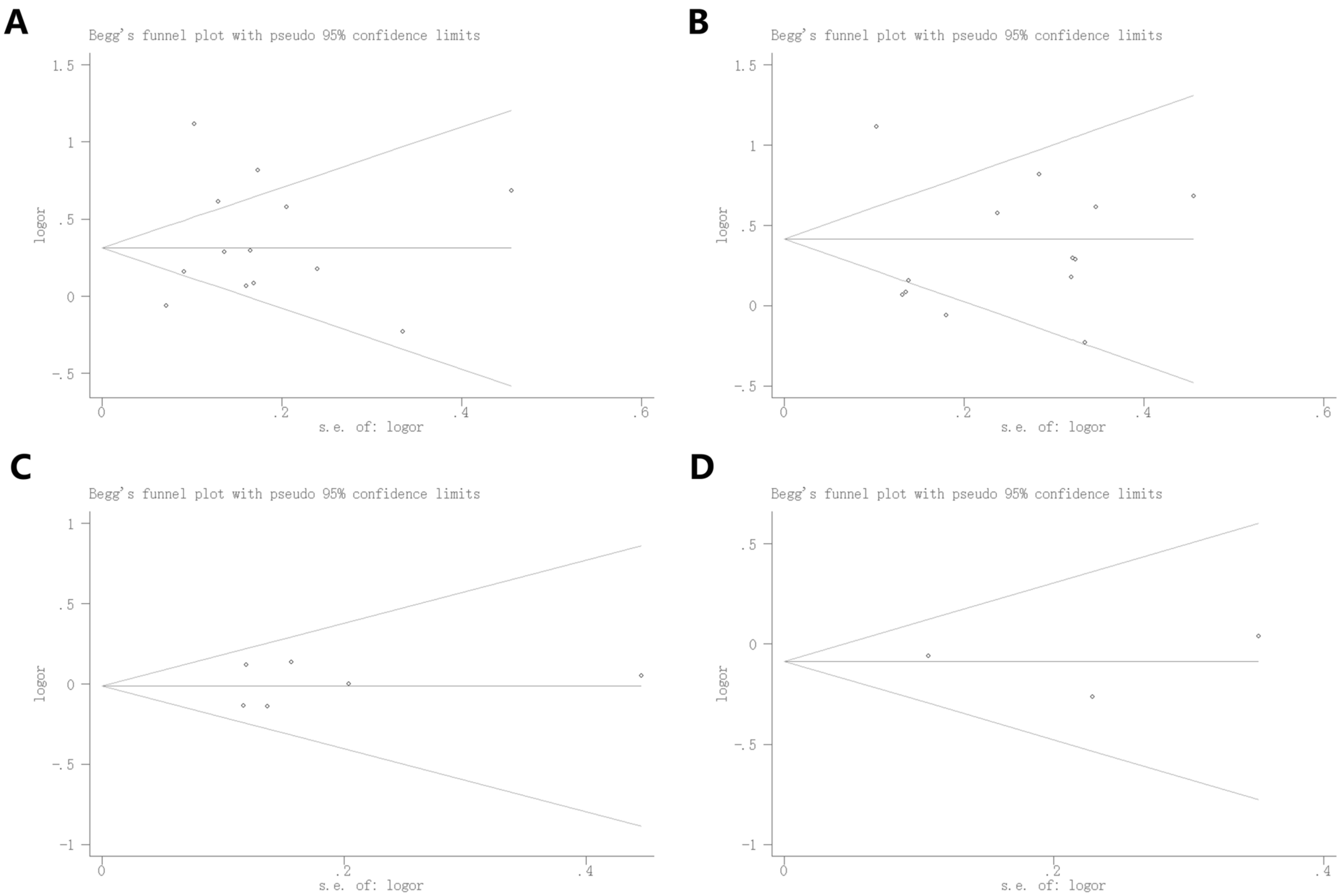

Figure S1. Begg's test for publication bias (dominant model). A rs4588; **B** rs7041; **C** rs3782130; **D** rs4646537

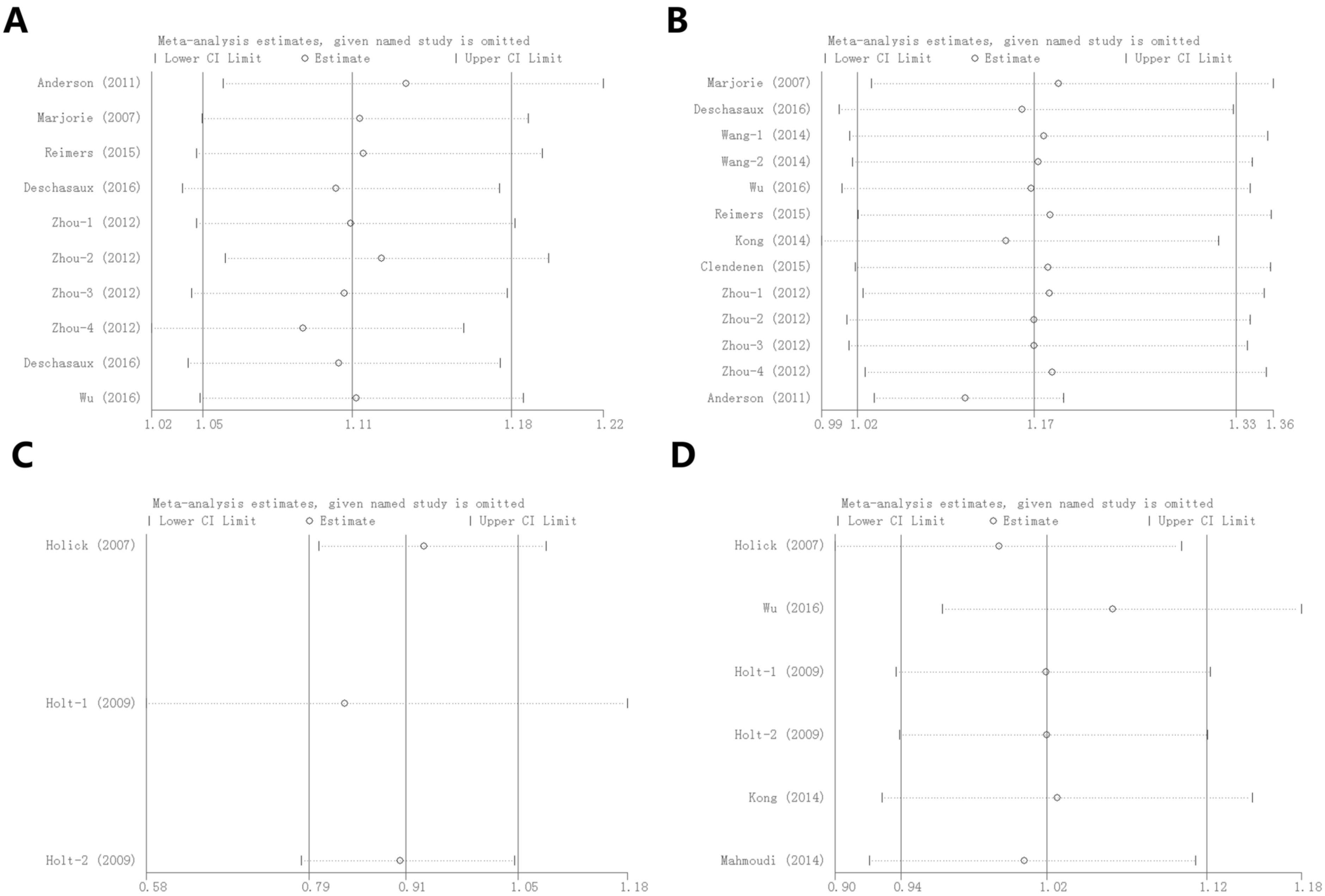

Figure S2. Sensitivity analyses of the studies (allele model). A rs4588; **B** rs7041; **C** rs4646537; **D** rs3782130
